# Sitagliptin ameliorates microbial dysbiosis and enhances gut barrier integrity in streptozotocin-induced type 2 diabetic rats

**DOI:** 10.3389/fmicb.2025.1655522

**Published:** 2025-09-19

**Authors:** Ying Chang, Yuanjiang Zhou, Fanlin Zhou, Jie Liang, Yu Li, Mingyuan Tian

**Affiliations:** ^1^Department of Geriatrics, Chongqing General Hospital, Chongqing University, Chongqing, China; ^2^Department of Endocrinology, The Second Affiliated Hospital of Chongqing Medical University, Chongqing, China; ^3^Department of Pathology, Chongqing Medical University, Chongqing, China; ^4^Department of Pathology, Chongqing University Cancer Hospital & Chongqing Cancer Institute & Chongqing Cancer Hospital, Chongqing, China; ^5^Institute of Pathology and Southwest Cancer Center, First Affiliated Hospital, Army Medical University, Chongqing, China; ^6^Department of Pathology, Chongqing Traditional Chinese Medicine Hospital, Chongqing, China

**Keywords:** sitagliptin, type 2 diabetes mellitus, gut microbiota, gut microbial network, intestinal barrier function

## Abstract

**Background:**

Sitagliptin, a dipeptidyl peptidase-4 (DPP-4) inhibitor, has demonstrated efficacy in the management of type 2 diabetes mellitus (T2DM). This study aimed to investigate the effects of sitagliptin on gut microbial composition and gut barrier integrity in a streptozotocin (STZ)-induced rat model of T2DM.

**Methods:**

Sprague-Dawley rats were randomly divided into four groups (*n* = 6 per group): a T2DM group induced by high-fat diet (HFD) and STZ injection; a T2DM group treated with oral sitagliptin at 10 mg/kg/day for 12 weeks (T2DM-Sit); a normal diet control group (ND); and a normal diet group treated with sitagliptin (ND-Sit). Fecal samples were collected for 16S rRNA gene sequencing to analyze gut microbial composition and diversity. Intestinal tissues were assessed for tight junction protein expression via immunohistochemistry and western blot.

**Result:**

Alpha index metrics, including observed feature number and Shannon index, were significantly decreased in the T2DM group compared to the ND group. Sitagliptin treatment led to a significant restoration of these indices. Principal coordinate analysis based on Bray-Curtis distances revealed distinct clustering between the ND and T2DM groups, with sitagliptin shifting the microbial profile of diabetic rats toward that of healthy controls. Sitagliptin treatment increased the relative abundance of *Lactobacillus, Ruminococcus, Streptococcus, Klebsiella, Clostridium_IV*, and *Romboutsia*, while reducing levels of *Alloprevotella* and *Parasutterella*, effectively reversing the dysbiotic changes observed in T2DM. Additionally, sitagliptin modulated microbial metabolic pathways, restructured the gut microbial network, and promoted short-chain fatty acid production. Histological and western blot analysis revealed enhanced expression of the tight junction protein ZO-1 and increased numbers of mucin-secreting goblet cells, indicating improved gut barrier integrity.

**Conclusion:**

Sitagliptin effectively ameliorates gut microbial dysbiosis and restores intestinal barrier function in STZ-induced T2DM rats. These findings provide novel insights into the gut-related therapeutic effects of sitagliptin and underscore its potential in restoring gut homeostasis in T2DM.

## Introduction

Type 2 diabetes mellitus (T2DM) has experienced a marked global increase in prevalence in recent years, driven by a combination of environmental influences and genetic predispositions. One of the key mechanisms implicated in the pathogenesis of T2DM is dysfunction of the intestinal barrier, which leads to increased gut permeability (Kelly et al., 2015). This compromised barrier function facilitates the systemic translocation of microbiota-derived endotoxins, such lipopolysaccharide (LPS), which has been recognized as a potent trigger of insulin resistance and the development of T2DM (Cani et al., 2007). Consequently, strategies aimed at enhancing intestinal barrier integrity have garnered significant attention as potential therapeutic approaches in T2DM management. In parallel, gut microbiota dysbiosis has been closely associated with impaired gut barrier function, further exacerbating insulin resistance and metabolic dysregulation in T2DM (Wu et al., 2020). Certain microbial taxa capable of producing short-chain fatty acids (SCFAs), particularly butyrate, have demonstrated a protective role in maintaining gut barrier integrity and modulating glucose metabolism (Canfora et al., 2019). As a result, interventions such as dietary modification, fecal microbiota transplantation (FMT), and probiotic supplementation have been extensively explored for their capacity to restore gut microbial homeostasis and improve insulin sensitivity through modulation of the gut microbiota and reinforcement of intestinal barrier function (Régnier et al., 2021).

Sitagliptin, a well-established dipeptidyl peptidase-4 (DPP-4) inhibitor, is widely prescribed for the treatment of T2DM. Its primary mechanism of action involves inhibiting DPP-4 activity to prevent the degradation of glucagon-like peptide-1 (GLP-1), an incretin hormone secreted by intestinal L-cells (Bodnaruc et al., 2016), thereby enhancing insulin secretion, suppressing glucagon release, reducing hepatic gluconeogenesis, and improving insulin sensitivity (Ross and Ekoé, 2010). Beyond its glycemic regulatory role, accumulating evidence suggests that sitagliptin exerts extra-glycemic effects, particularly at the intestinal level (Ning et al., 2020). DPP-4 is highly expressed on the surface of intestinal epithelial and immune cells, where it plays a role in modulating local immune responses, maintaining epithelial barrier integrity, and interacting with the gut microbiota (Yasuda et al., 2024). Sitagliptin has been shown to influence the balance between apoptosis and proliferation in intestinal epithelial cells, reduce intestinal inflammation, and alleviate lipopolysaccharide-induced damage by inhibiting NF-κB signaling pathway (Lin et al., 2016). Recent studies further suggest that sitagliptin treatment may attenuate T2DM-associated gut microbial dysbiosis (Liao et al., 2019; Yan et al., 2016; Zhang et al., 2019). These findings collectively indicate that the intestine serves not only as a source of incretin secretion but also as a direct target organ for DPP-4 inhibitors, providing a plausible mechanistic link between sitagliptin therapy and improvements in gut health and microbial homeostasis.

The gut microbiome, comprising trillions of microorganisms and thousands of diverse species, functions as a complex and dynamic ecosystem. Microbial members interact through the exchange of metabolites, signaling molecules, and genetic material, engaging in intricate ecological networks that influence host physiology (Li et al., 2020; Layeghifard et al., 2017). Understanding these microbial interactions from a dynamic ecological perspective is essential for elucidating the functional contributions of the gut microbiome to health and disease (Cao et al., 2022; Chen et al., 2020). The microbiome is subject to continuous structural and functional shifts in response to internal and external perturbations, highlighting its adaptive complexity and the necessity for comprehensive approaches in microbiome research (Li et al., 2020; Sun et al., 2023).

Based on these considerations, we hypothesized that sitagliptin may ameliorate T2DM not only through systemic glucose regulation but also by modulating the gut microbiome and improving intestinal barrier integrity. To validate this hypothesis, we assessed tight junction protein expression and goblet cell abundance, which are established markers of gut barrier health, in parallel with gut microbial community profiling. This study aims to provide new insights into the broader biological effects of sitagliptin, particularly its influence on gut microbial ecology and gut integrity during the progression of T2DM.

## Material and methods

### Establishment of the diabetic rat model

Male Sprague-Dawley (SD) rats aged 6 to 8 weeks, weighing 110 ± 20 g, were obtained from the Experimental Animal Center of Chongqing Medical University. Animals were acclimated for 1 week under controlled environmental conditions: a 12-h light/dark cycle, relative humidity of 45 ± 10%, and temperature of 21 ± 2 °C. During this period, rats had ad libitum access to water and a standard laboratory chow diet (10% calories from fat). Following acclimatization, rats were randomly assigned to a control group (ND) or a high-fat diet (HFD) group. The ND group received a standard laboratory chow diet (10% fat, 20% protein, and 70% carbohydrates), while the HFD group received a high-fat diet (45% fat, 20% protein, and 35% carbohydrates; Medicine Ltd., Jiangshu, China) for 12 weeks. At the end of the dietary intervention, rats in the HFD group received an intraperitoneal injection of streptozotocin (STZ, 30 mg/kg; Sigma-Aldrich, Saint Louis, MO) dissolved in 0.1 mol/L citrate buffer (pH 4.5), while the ND group received an equal volume of citrate buffer. After 72 h following STZ injection, random blood glucose levels were measured from tail vein blood using a glucometer. The T2DM model was successfully replicated in rats with random blood glucose levels exceeding 16.7 mmol/L, according to previous report (Tawulie et al., 2023).

Of the 16 rats subjected to HFD+STZ treatment, 12 met the criteria for diabetes and were subsequently randomized into two groups: vehicle-treated T2DM (T2DM, *n* = 6) and sitagliptin-treated T2DM (T2DM-Sit, 10 mg/kg/day, *n* = 6). Similarly, ND rats were randomly divided into ND (*n* = 6) and sitagliptin-treated ND (ND-Sit, 10 mg/kg/day, *n* = 6, one sample was excluded due to insufficient DNA). Sitagliptin (10 mg/kg/day) was administered by oral gavage once daily for 12 weeks following established protocols (Yan et al., 2016). Both the ND and T2DM groups continued their respective diets for an additional 12 weeks. Fresh fecal samples were collected after 12 weeks of sitagliptin treatment and stored at −80 °C for further analysis. At the end of the experiment, all rats were anesthetized using 1% sodium pentobarbital (50 mg/kg, i.p.) and euthanized by cervical dislocation.

All animal protocols were approved by the Animal Ethics Committee of Animal Center of Chongqing Medical University and conducted in accordance with the institutional Guidelines for the Care and Use of Laboratory Animals.

### Fasting blood glucose and body weight measurement

Fasting blood glucose was measured using a Bayer Contour TS glucometer and test strips (Bayer, Hamburg, Germany). Body weight was recorded post-treatment for all rats.

### Fecal DNA extraction, sequencing, and bioinformatics analysis

Microbial DNA was extracted from fecal samples using the QIAamp Fast DNA Stool Mini Kit (Qiagen), following the manufacturer's protocol. The V3-V4 hypervariable regions of the bacterial 16S rRNA gene were amplified using primer 341F (5′-CCTACGGGNGGCWGCAG-3′) and 806R (5′-GACTACHVGGGTATCTAATCC-3′). PCR products were purified using the QIAquick PCR Purification Kit, pooled in equal concentrations and sequenced on an Illumina MiSeq platform using a paired-end read mode.

Sequencing quality was assessed using FastQC, and high-quality reads were processed using Mothur (Schloss et al., 2009). Paired-end reads were assembled and aligned to the SILVA 119 reference database (Quast et al., 2013). Reads containing ambiguous bases or misalignments were discarded. Chimeric sequences were identified and removed using UCHIME in reference mode (Edgar et al., 2011). The remaining sequences were clustered into operational taxonomic units (OTUs) at 97% similarity. Taxonomic assignment was conducted using the Ribosomal Database Project (RDP) Naive Bayesian Classifier with the RDP training set (version 10) (Wang et al., 2007). Functional profiling of microbial metabolic pathways was performed using PICRUSt2.0 software (Douglas et al., 2020). Microbial co-occurrence networks were constructed using the R package ggClusterNet to evaluate microbial interactions (Wen et al., 2022).

### Quantification of short-chain fatty acids in fecal samples

Short-chain fatty acids (SCFAs) were quantified using gas chromatography–mass spectrometry (GC-MS). Standard stock solution A was prepared by dissolving eight SCFAs (acetic, butyric, hexanoic, isobutyric, isohexanoic, isovaleric, propionic, and valeric acids) in HPLC-grade *n*-butanol. Internal standard solution B consisted of 2-ethylbutyric acid in *n*-butanol. Both were serially diluted to generate a seven-point calibration curve.

For sample preparation, 25 mg of feces was homogenized with 500 μL of 0.5% phosphoric acid solution at 50 Hz, followed by ultrasonication and centrifugation (13,000 g, 4 °C, 15 min). The supernatant (200 μL) was extracted with *n*-butanol containing 10 μg/mL internal standard.

Analysis was performed using an Agilent 8890B GC system coupled with a 5977B/7000D MS and equipped with an HP-FFAP capillary column (30 m × 0.25 mm × 0.25 μm). The oven temperature was programmed to increase from 80 °C to 120 °C at 40 °C/min, then to 200 °C at 5 °C/min, and held at 220 °C for 3 min. Data acquisition and compound quantification were performed using MassHunter software. Quality control samples were run every 5–10 samples to ensure system consistency.

### Histological and immunohistochemical analysis

Colons were harvested, rinsed with PBS, fixed overnight in 4% formaldehyde, and embedded in paraffin. Sections of 4 μm thickness were prepared and stained with hematoxylin and eosin (H&E) or Alcian blue-periodic acid–Schiff (AB-PAS) stain to visualize goblet cells, which were stained blue. Goblet cell density was quantified as the number of goblet cells per intestinal villus.

Immunohistochemical staining was performed using a modified streptavidin–HRP method (CoWin Century Biotechnology, China). Briefly, paraffin sections were deparaffinized, endogenous peroxidase activity was quenched using 0.3% H_2_O_2_, and antigen retrieval was carried out via microwave treatment in 10 mmol/L citrate buffer (pH 6.0). Sections were blocked with normal goat serum at 37 °C for 30 min and incubated overnight with primary antibodies against ZO-1 (Abcam, ab276131) and Claudin-1 (Abcam, ab307692). After incubation with HRP-conjugated streptavidin, signals were visualized using a DAB detection kit (CoWin Century Biotechnology, China) and counterstained with hematoxylin. Positive cell counts were quantified using ImageJ software.

### Western blotting analysis

Protein concentrations were determined using the bicinchoninic acid (BCA) assay. Equal amounts of total protein (20 μg per lane) were separated by 8–10% sodium dodecyl sulfate-polyacrylamide gel electrophoresis (SDS-PAGE) and subsequently transferred onto polyvinylidene fluoride (PVDF) membranes. After transfer, the membranes were blocked with 5% skim milk in TBST for 2 h at room temperature and then incubated overnight at 4 °C with the following primary antibodies: anti-ZO-1 (1:1000, ab276131, Abcam), anti-Claudin (1:1000, ab307692, Abcam), anti-IL-1β (1:1000, TA384520M, Origene), anti-IL-6 (1:1000, TA384528S, Origene), anti-NF-κB (1:2000, TA890002M, Origene), and anti-β-actin (1:1000, PMK081M/S, Bioprimacy).

After washing with TBST, membranes were incubated with appropriate horseradish peroxidase (HRP)-conjugated secondary antibodies for 1 h at room temperature. Protein bands were visualized using the Immobilon^®^ Western Chemiluminescent HRP Substrate (Millipore), and signal intensities were quantified using ImageJ software (NIH, USA). Densitometric values of target proteins were normalized to β-actin.

### Statistical analysis

Continuous variables are expressed as means ± standard deviations and were compared using Student's *t*-test. Categorical data were analyzed using the chi-square test. Differences in gut microbial community composition were assessed using principal coordinates analysis (PCoA), an unsupervised ordination method used for dimensionality reduction and visualization of beta diversity. Statistical significance of group-wise differences was evaluated using permutational multivariate analysis of variance (PERMANOVA) with 999 permutations. These analyses were performed in R software with a significance level set at *P* < 0.05. Differentially abundant taxa between groups were identified using LEfSe (Linear Discriminant Analysis Effect Size), with significance defined as an LDA score > 2.0 and *P* < 0.05 (Segata et al., 2011). Functional pathway differences were analyzed using STAMP software with Bonferroni correction applied (Parks et al., 2014).

## Results

### Body weight and fasting blood glucose

As shown in Table 1, the body weight of rats in the T2DM group was significantly higher than that of the ND group (535.7 ± 26.54 g vs. 347.53 ± 15.96 g, *P* < 0.01). Fasting blood glucose levels were also markedly elevated in the T2DM group compared to the ND group (20.85 ± 1.68 mmol/L vs. 5.79 ± 0.64 mmol/L, *P* < 0.01). Treatment with sitagliptin significantly reduced blood glucose levels in the T2DM-Sit group compared to the untreated T2DM group (15.53 ± 1.94 mmol/L vs. 20.85 ± 1.68 mmol/L, *P* < 0.01), although no significant difference in body weight was observed between these two groups (507.45 ± 16.43 g vs. 535.7 ± 26.54 g, *P* = 0.051). Furthermore, there were no statistically significant differences in either body weight (347.53 ± 15.96 g vs. 330.5 ± 21.48 g, *P* = 0.17) or blood glucose levels (5.79 ± 0.64 mmol/L vs. 4.98 ± 0.88 mmol/L, *P* = 0.11) between the ND and ND-Sit groups.

**Table 1:** Body weight and fasting blood glucose changes during progression of glucose intolerance and after sitagliptin treatment.

**Group**	**Body weight (grams)**	**Blood glucose (mmol/L)**
ND (*n* = 6)	347.53 ± 15.96^**^	5.79 ± 0.64^**^
ND-Sit (*n* = 5)	330.5 ± 21.48	4.98 ± 0.88
T2DM (*n* = 6)	535.7 ± 26.54	20.85 ± 1.68
T2DM-Sit (*n* = 6)	507.45 ± 16.43	15.53 ± 1.94^**^

Data are presented as mean ± SD. ^*^ indicates *P* < 0.05, and ^**^ indicates *P* < 0.01 compared to the T2DM group.

### Reversal of gut microbial diversity alterations in response to sitagliptin treatment

**Figure 1:**
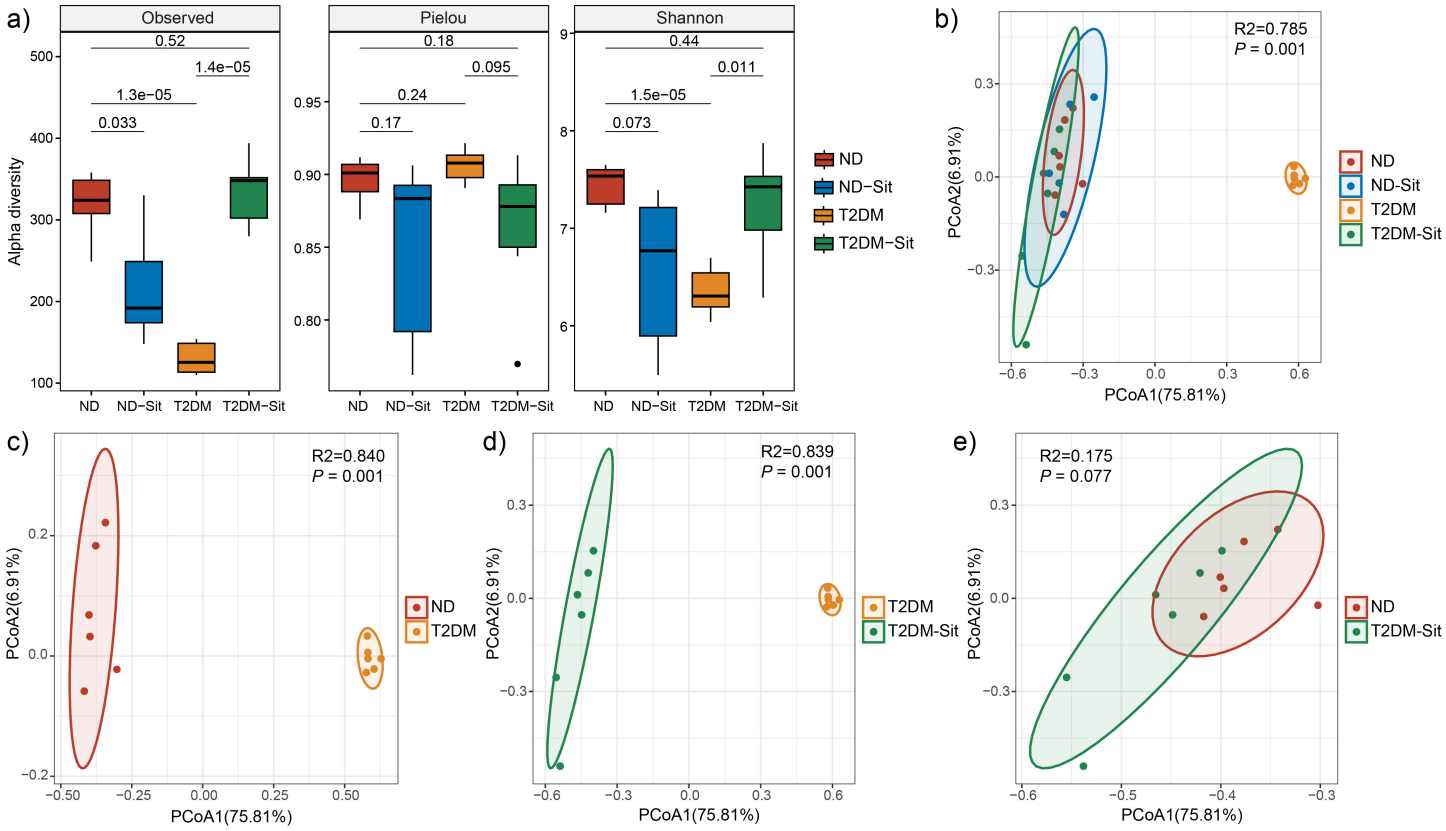
Comparisons of alpha and beta diversity among the ND, ND-Sit, T2DM and T2DM-Sit groups. **(a)** Alpha diversity indices, including observed feature number, Pielou index, and Shannon index. **(b)** PCoA plot based on Bray–Curtis dissimilarity among all four groups. **(c)** PCoA plot between the ND and T2DM groups. **(d)** PCoA plot between the T2DM and T2DM-Sit groups**. (e)** PCoA plot between the ND and T2DM-Sit groups.

Alpha diversity was assessed using observed feature numbers, Pielou's evenness, and the Shannon index (Figure 1a). Compared to the ND group, both the ND-Sit (*P* = 0.033) and T2DM (*P* < 0.01) groups exhibited significantly reduced observed feature numbers. Notably, the T2DM-Sit group demonstrated a significant increase in observed feature numbers relative to the T2DM group (*P* < 0.01). Similarly, Shannon index values showed a downward trend in the ND-Sit group compared to the ND group, though the difference was not statistically significant. A significant decrease was observed in the T2DM group compared to ND (*P* < 0.01), while the T2DM-Sit group exhibited a significant increase in Shannon index compared to the T2DM group (P = 0.011). No significant differences were observed in the Pielou index among the four groups.

Beta diversity analysis using Bray-Curtis distances revealed significant separation among the ND-Sit, T2DM, and T2DM-Sit groups based on PERMANOVA (Figure 1b). Distinct differences in microbial community composition were observed between the ND and T2DM groups (*R*^2^ = 0.84, *P* = 0.001, Figure 1c), and between the T2DM and T2DM-Sit groups (*R*^2^ = 0.839, *P* = 0.001, Figure 1d). Notably, no significant difference was observed between the ND and T2DM-Sit groups (*R*^2^ = 0.175, *P* = 0.077, Figure 1e), suggesting that sitagliptin treatment may help restore the gut microbial structure toward a healthy state.

### Reversal of gut microbial composition in response to sitagliptin treatment

**Figure 2:**
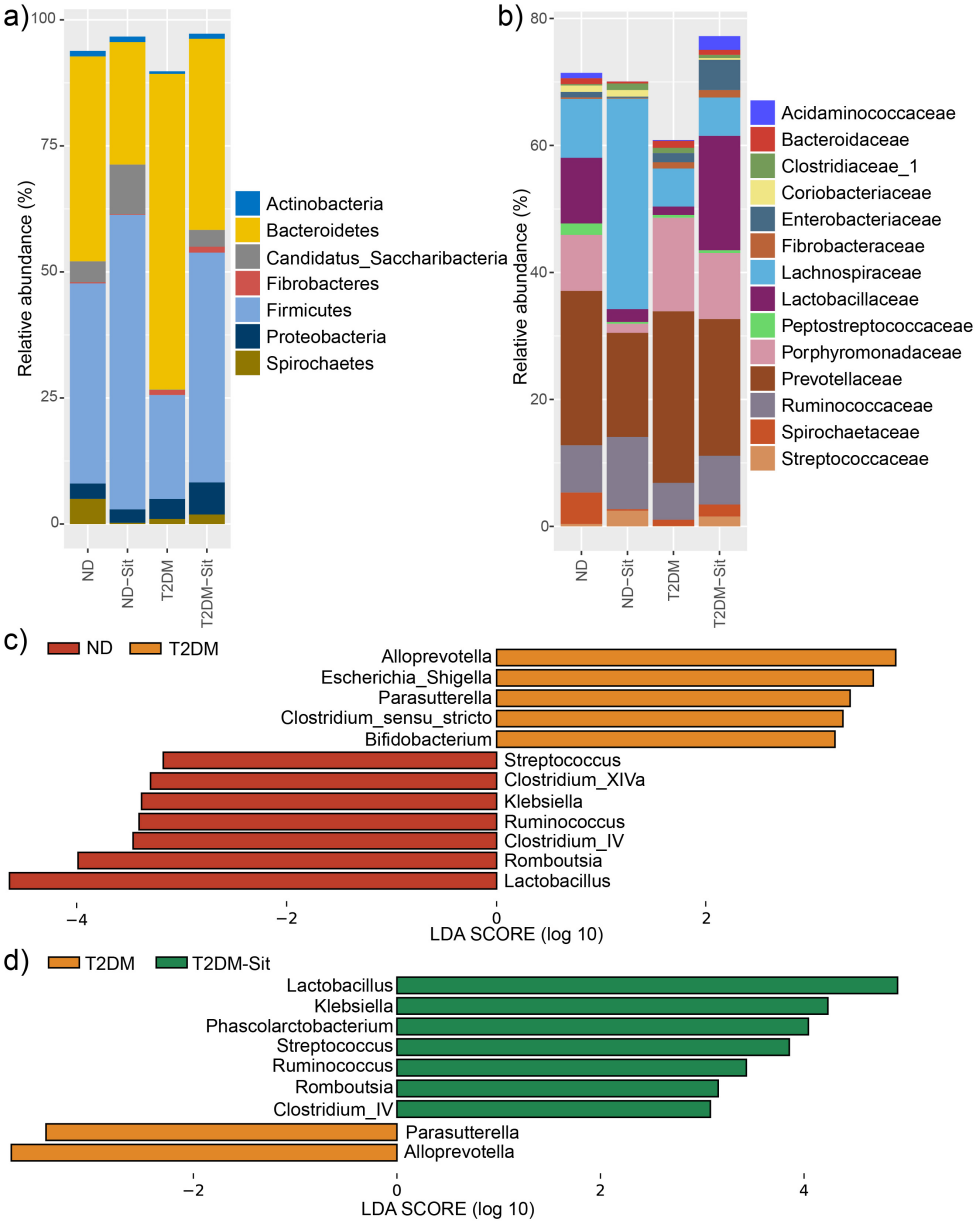
Relative abundances of abundant microbes and their differences among the ND, ND-Sit, T2DM, and T2DM-Sit groups. **(a)** The average relative abundances of abundant phyla of the 4 groups. **(b)** The average relative abundance of abundant families of the 4 groups. **(c)** The significantly different genera between the ND and T2DM groups. **(d)** The significantly different genera between the T2DM and T2DM-Sit groups.

A total of 2,271,414 high-quality sequencing reads were obtained across all 23 samples, averaging 98,757 reads per sample. The dominant phyla included *Actinobacteria, Bacteroidetes, Candidatus_Saccharibacteria, Fibrobacteres, Firmicutes, Proteobacteria*, and *Spirochaetes* (Figure 2a). Compared to the ND group, the T2DM group exhibited an increase in the relative abundance of *Bacteroidetes* and a concomitant decrease in *Firmicutes*. Treatment with sitagliptin restored this imbalance, resulting in a reduction of *Bacteroidetes* and an elevation of *Firmicutes*, thereby shifting the microbial composition toward that observed in the ND group. At the family level, the main taxa identified were *Acidaminococcaceae, Bacteroidaceae, Clostridiaceae_1, Coriobacteriaceae, Enterobacteriaceae, Fibrobacteraceae, Lachnospiraceae, Lactobacillaceae, Peptostreptococcaceae, Porphyromonadaceae, Prevotellaceae, Ruminococcaceae, Spirochaetaceae*, and *Streptococcaceae* (Figure 2b).

Further, at the genus level, LEfSe analysis (average relative abundance > 0.1%) was performed to identify significant differences among the four groups. The T2DM group showed increased abundance of *Alloprevotella, Escherichia_Shigella, Parasutterella, Clostridium_sensu_stricto*, and *Bifidobacterium*, while *Streptococcus, Clostridium_XIVa, Klebsiella, Ruminococcus, Clostridium_IV, Romboutsia*, and *Lactobacillus* were significantly decreased compared to the ND group (Figure 2c). After sitagliptin treatment, the T2DM-Sit group exhibited increased abundances of *Lactobacillus, Klebsiella, Phascolarctobacterium, Streptococcus, Ruminococcus, Romboutsia*, and *Clostridium_IV*, alongside decreased abundances of *Parasutterella* and *Alloprevotella* (Figure 2d). To further explore the relationship between microbial alterations and glycemic control, Pearson correlation analysis was conducted. Notably, the abundance of *Escherichia_Shigella* and *Parasutterella* showed significant positive correlations with blood glucose levels (*r* = 0.44 and *r* = 0.47, respectively; *P* < 0.05), suggesting that reductions of these taxa may contribute to the glucose-lowering effects of sitagliptin. These findings suggest that sitagliptin reversed diabetes-associated dysbiosis.

### Reversal of metabolic functional pathways in response to sitagliptin treatment

**Figure 3:**
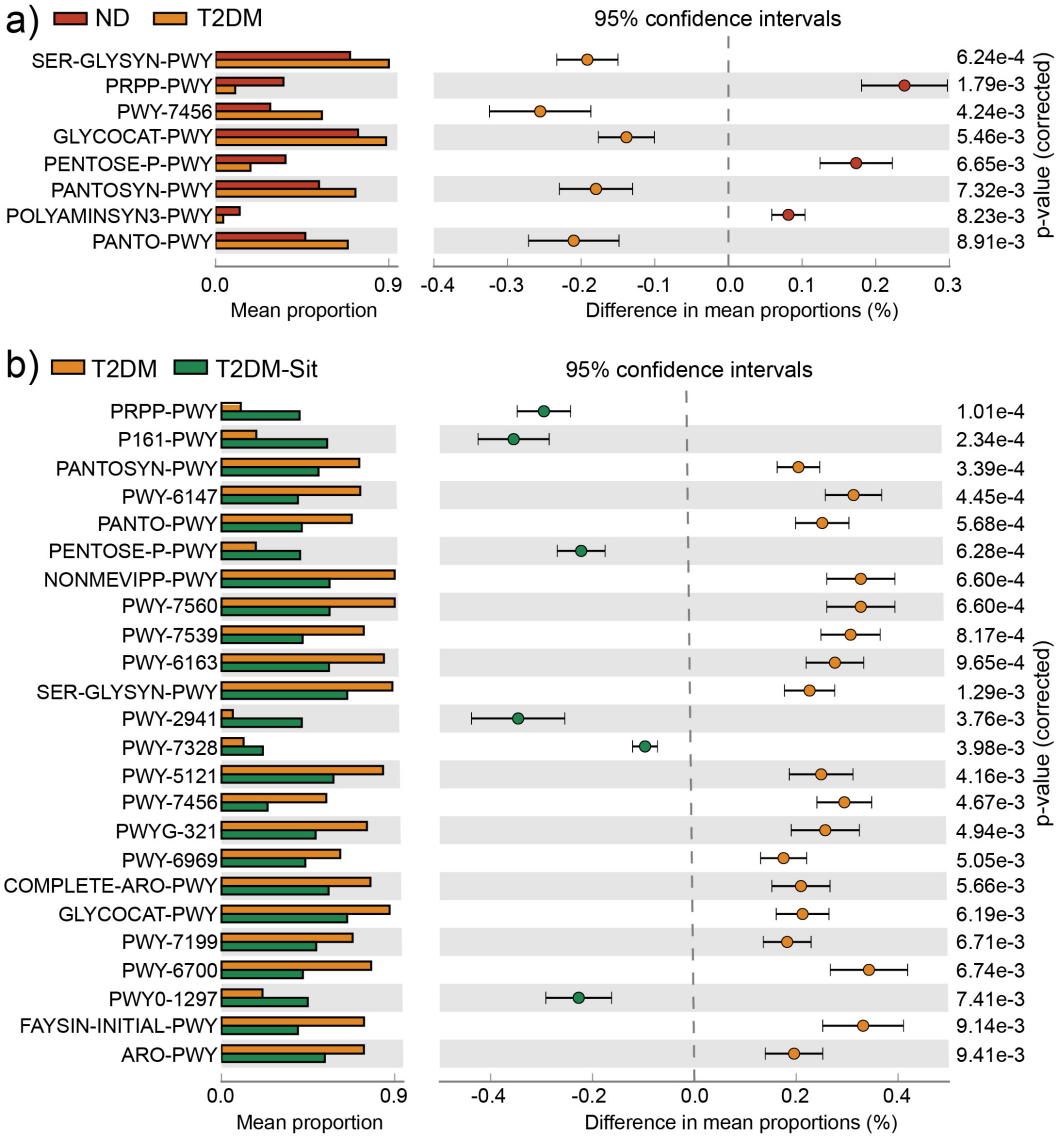
Functional metabolic pathways with significantly differential abundance among the ND, ND-Sit, T2DM, and T2DM-Sit groups. **(a)** Different metabolic pathways between the ND and T2DM groups. **(b)** Different metabolic pathways between the T2DM and T2DM-Sit groups.

Functional predictions based on PICRUSt2.0 and the MetaCyc database, analyzed via STAMP, revealed eight significantly different metabolic pathways between the ND and T2DM groups (Figure 3a). These included the “superpathway of L-serine and glycine biosynthesis I (SER-GLYSN-PWY)”, “superpathway of histidine, purine, and pyrimidine biosynthesis (PRPP-PWY)”, “β-(1,4)-mannan degradation (PWY-7456)”, “glycogen degradation I (GLYCOCAT-PWY)”, “pentose phosphate pathway (PENTOSE-P-PWY)”, “superpathway of coenzyme A biosynthesis I (PANTOSYN-PWY)”, “superpathway of polyamine biosynthesis II (POLYAMINSYN3-PWY)”, and “phosphopantothenate biosynthesis I (PANTO-PWY)”. The PRPP-PWY and PENTOSE-P-PWY pathways were more abundant in the ND group, whereas the remaining six pathways were enriched in the T2DM group. Following sitagliptin treatment, the T2DM-Sit group exhibited increased abundances of PRPP-PWY and PENTOSE-P-PWY, along with reduced abundances in the other six pathways (Figure 3b), indicating a metabolic shift toward the healthy ND profile. Additionally, the T2DM-Sit group showed more differential pathways compared to the T2DM group, suggesting a broad modulatory effect of sitagliptin on microbial metabolism.

### Reversal of microbial network interactions in response to sitagliptin treatment

**Figure 4:**
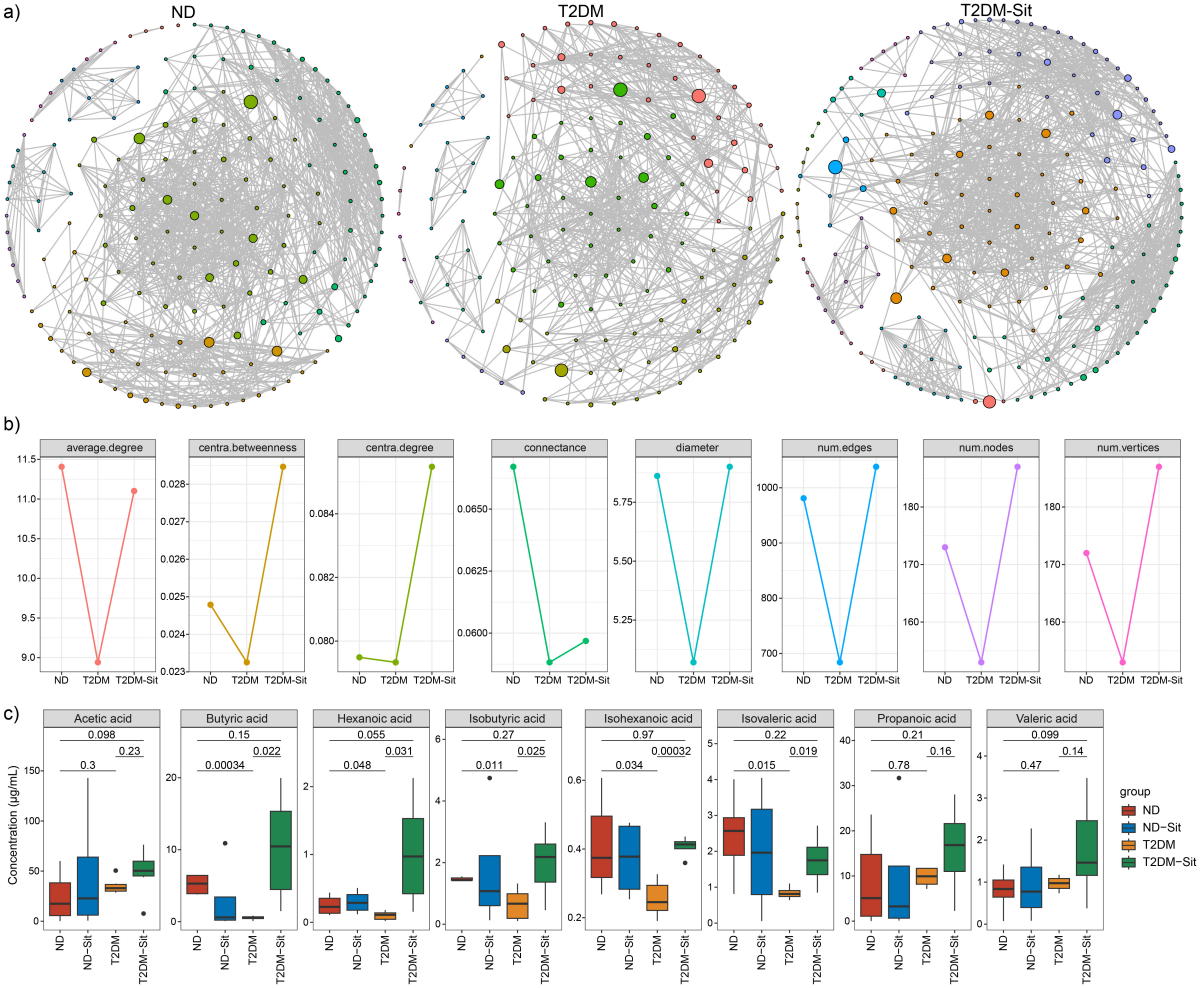
Gut microbial network of the ND, T2DM and T2DM-Sit groups. **(a)** The gut microbial network in the ND, T2DM, T2DM-Sit group separately. **(b)** The comparisons of gut microbial networks' parameters among the three groups. **(c)** The comparisons of SCFAs among the ND, T2DM, T2DM-Sit groups.

Microbial co-occurrence networks were constructed to explore community interactions within the ND, T2DM, and T2DM-Sit groups (Figure 4a). The ND group exhibited a network with 173 nodes, 981 edges, and a connectance of 0.067, average degree of 11.41, diameter of 5.86, centralization degree of 0.080, and centralization betweenness of 0.025. In contrast, the T2DM group showed a simpler network with 153 nodes, 684 edges, a connectance of 0.059, average degree of 8.94, diameter of 5.07, centralization degree of 0.079, and centralization betweenness of 0.023, indicating reduced microbial connectivity and complexity (Figure 4b). Following sitagliptin treatment, the T2DM-Sit group displayed a more robust network with 187 nodes, 1,038 edges, a connectance of 0.060, average degree of 11.10, diameter of 5.90, centralization degree of 0.086, and centralization betweenness of 0.029, suggesting restoration of microbial interactions. These results highlight the disruption of microbial network structure in T2DM and its normalization upon sitagliptin intervention.

### Reversal of SCFAs in response to sitagliptin treatment

Short-chain fatty acids (SCFAs) were quantified via GC-MS, including acetic, butyric, hexanoic, isobutyric, isohexanoic, isovaleric, propanoic, and valeric acids. The T2DM group exhibited significantly lower levels of butyric, hexanoic, isobutyric, isohexanoic, and isovaleric acids compared to the ND group (Figure 4c). Sitagliptin treatment reversed these reductions, as the T2DM-Sit group showed increased levels of these SCFAs, suggesting a restorative effect of sitagliptin on microbial metabolite production.

### Sitagliptin improves intestinal barrier integrity

**Figure 5:**
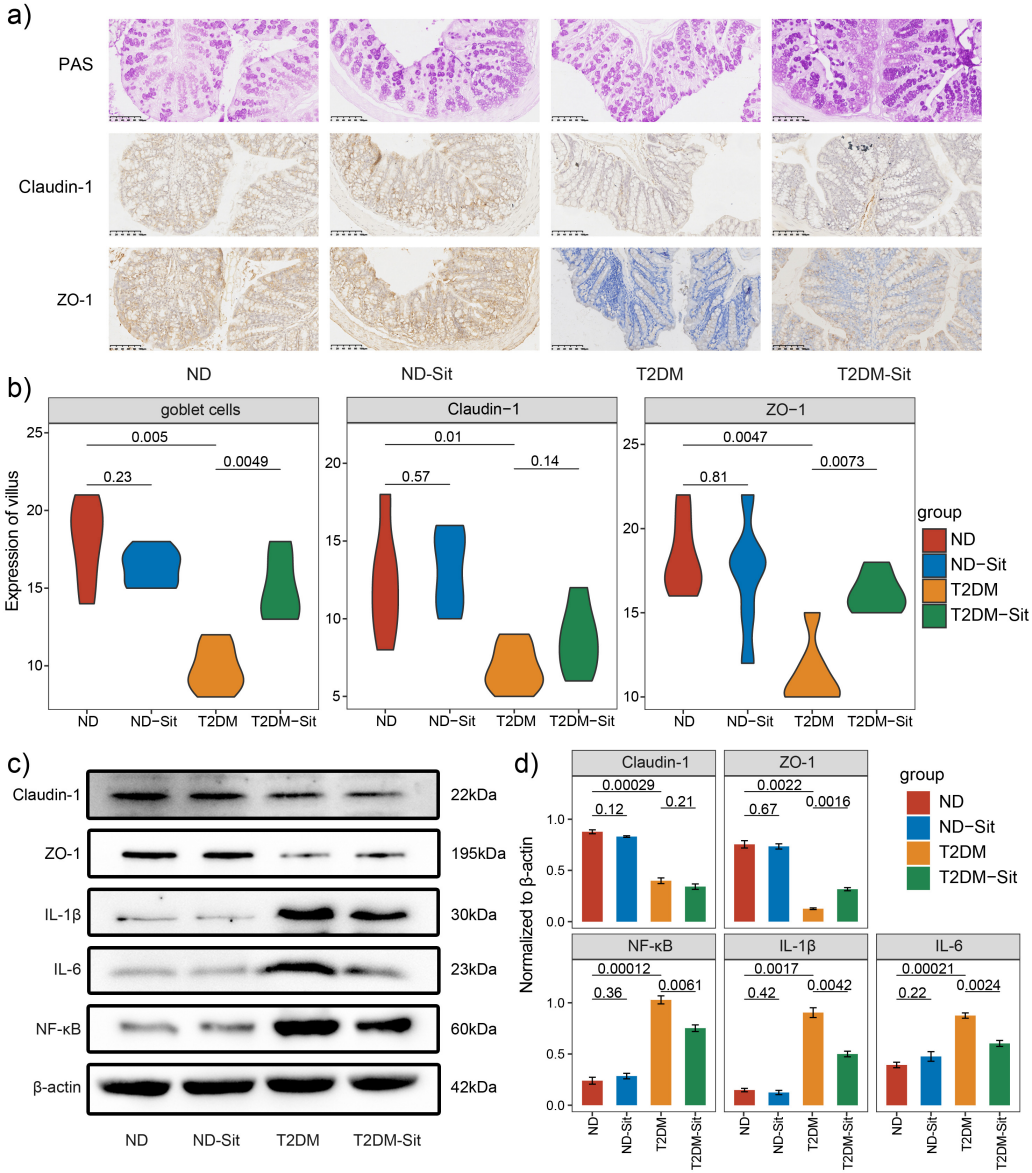
Sitagliptin enhances gut barrier integrity in diabetic rats. **(a)** Representative images of colonic tissue stained with Alcian Blue–Periodic Acid-Schiff (AB-PAS) staining. **(b)** Quantification of goblet cell numbers and expression levels of Claudin-1 and ZO-1 in colonic tissues. **(c)** Representative Western blot images showing the protein expression of Claudin-1, ZO-1, NF-κB, IL-1β, and IL-6, with β-actin used as the loading control. **(d)** Densitometric analysis of Western blot bands for Claudin-1, ZO-1, NF-κB, IL-1β, and IL-6 across different groups. Protein expression was normalized to β-actin.

To investigate the effects of sitagliptin on intestinal barrier integrity, histological and western blot analyses were performed on colonic tissues. PAS-Alcian blue staining revealed an increased number of mucus-secreting goblet cells in the T2DM-Sit group compared to the T2DM group (Figure 5a), indicating improved mucosal protection following sitagliptin treatment.

Immunohistochemical staining was conducted to assess the expression of tight junction proteins ZO-1 and Claudin-1. Compared with the ND group, the T2DM group showed a significant reduction in ZO-1- and Claudin-1-positive cells. Notably, sitagliptin treatment markedly restored ZO-1 expression, whereas Claudin-1 levels increased without significance (Figure 5b). These findings were further corroborated by western blot analysis, which demonstrated that both ZO-1 and Claudin-1 protein levels were significantly reduced in the T2DM group relative to the ND group. Consistent with the immunohistochemistry results, sitagliptin administration significantly upregulated ZO-1 expression, while Claudin-1 expression remained unaffected (Figures 5c, d).

In addition, western blot analysis revealed that proinflammatory markers NF-κB, IL-1β, and IL-6 were significantly elevated in the T2DM group but were markedly suppressed following sitagliptin treatment (Figures 5c, d). Interestingly, no significant differences in the expression of tight junction proteins or inflammatory cytokines were observed between the ND and ND-Sit groups, suggesting that sitagliptin does not interfere with normal gut homeostasis. Collectively, these results suggest that sitagliptin may exert a protective effect on intestinal barrier function in type 2 diabetic rats by enhancing ZO-1 expression and attenuating inflammation.

## Discussion

Accumulating evidence has demonstrated that T2DM is associated with impaired gut barrier function and dysbiosis of the gut microbiota. Sitagliptin, one of the most commonly prescribed medications for T2DM, has been extensively studied for its glycemic regulatory effects. In our study, sitagliptin administration significantly reduced blood glucose levels in diabetic rats although not to normoglycemic levels, consistent with previous findings (Yan et al., 2016), which might be attributed to the mechanism of DPP-4 inhibitors. Importantly, this modest glycemic improvement was accompanied by substantial amelioration of gut microbiota composition and intestinal barrier integrity, suggesting that sitagliptin may exert therapeutic benefits beyond glucose regulation.

The gut barrier plays a crucial role in maintaining intestinal homeostasis by acting as a selective filter, degrading pathogens and antigens, preventing microbial adhesion and colonization, and initiating appropriate immune responses. Tight junctions, composed of transmembrane proteins such as claudins and cytoplasmic scaffolding proteins like ZO-1, are essential for the regulation of intestinal permeability (Chelakkot et al., 2018). In T2DM, the gut barrier is frequently compromised, leading to increased intestinal permeability and inflammation (Murugesan et al., 2025). In our study, diabetic rats exhibited a marked decrease in ZO-1 protein expression and goblet cell numbers, both of which are indicative of compromised intestinal barrier function. Sitagliptin treatment significantly restored ZO-1 expression and increased goblet cell abundance, suggesting a protective effect on epithelial integrity. These observations are supported by previous reports that DPP-4 inhibitors can enhance gut barrier function (Yasuda et al., 2024). These findings indicate that the protective effect of sitagliptin on gut barrier function may be closely associated with the restoration of epithelial tight junctions.

The gut microbiota is an integral component of the intestinal barrier and plays a pivotal role in the maturation of mucosal barrier function, immune system development, nutrient absorption, and energy metabolism. In this study, we investigated the impact of sitagliptin on the intestinal microbiota of diabetic rats using 16S rRNA gene sequencing targeting the V3–V4 region. Diabetic rats exhibited significantly reduced alpha microbial diversity compared to the normal diet (ND) group, a finding consistent with previous reports (Yan et al., 2016). Sitagliptin treatment significantly increased microbial diversity in the diabetic group (T2DM-Sit), indicating a restorative effect on microbial richness and evenness. PCoA further confirmed distinct microbial community structures among the ND, T2DM, and T2DM-Sit groups, aligning with previous findings (Liao et al., 2019; Yan et al., 2016). The T2DM group showed clear separation from the ND group, whereas the microbial profiles of the T2DM-Sit group closely resembled those of the ND group, with substantial overlap between the two. This suggests that sitagliptin treatment may help normalize gut microbial composition in diabetic conditions.

Consistent with previous studies (Liao et al., 2019; Yan et al., 2016), Bacteroidetes and Firmicutes were the dominant phyla across all groups. Diabetic rats exhibited a notable increase in the relative abundance of the genera *Alloprevotella* and *Parasutterella*, alongside a decrease in *Streptococcus, Klebsiella, Ruminococcus, Clostridium_IV, Romboutsia*, and *Lactobacillus*. Sitagliptin treatment reversed these trends, significantly reducing *Alloprevotella* and *Parasutterella* while increasing the abundance of the aforementioned beneficial genera. Previous research has associated elevated *Alloprevotella* levels with prediabetic states (Pinna et al., 2021), and *Parasutterella* has been implicated in gut dysbiosis in diabetic models (Fernandez-Quintela et al., 2023). Reduction in *Lactobacillus*, a genus known for its probiotic effects, have been reported in T2DM (Yan et al., 2016; Fernandez-Quintela et al., 2023). Specific species of *Lactobacillus* have been linked to improvements in glucose and lipid metabolism, oxidative stress, inflammatory modulation, and gut barrier function, partly through downregulation of pro-inflammatory cytokines such as *TNF-*α (Wang et al., 2018; Yan et al., 2019). Similarly, *Ruminococcus* depletion has been observed in diabetic patients (Yan et al., 2016; Cunningham et al., 2021), and supplementation with *Streptococcus thermophilus* has been shown to lower fasting glucose and cholesterol levels in diabetic models (Gao et al., 2019). Multistrain probiotic formulations containing *Lactobacillus acidophilu*s, *S. thermophilus, L. bulgaricus* have demonstrated efficacy in improving glycemic control in T2DM patients (Tiderencel et al., 2020). Furthermore, *Romboutsia* has been suggested to support insulin sensitivity and metabolic regulation (Chen et al., 2021). Short-chain fatty acids, especially butyrate, play a crucial role in maintaining gut and metabolic balance. These metabolites contribute to improved insulin sensitivity, reduced inflammation, and gut barrier integrity (Salamone et al., 2021; Martin-Gallausiaux et al., 2021; Arora and Tremaroli, 2021). Notably, genera such as *Lactobacillus* and *Ruminococcus* are prominent SCFA producers (Fusco et al., 2023). In our study, levels of SCFAs, including butyric, hexanoic, isobutyric, isohexanoic, and isovaleric acids, were significantly reduced in the T2DM group, but were restored following sitagliptin treatment. SCFAs, particularly butyrate, serve as primary energy sources for colonocytes and have been shown to promote the expression of tight junction proteins such as ZO-1, occludin, and claudins, thereby enhancing epithelial integrity (Parada Venegas et al., 2019; Peng et al., 2009). These findings underscore the importance of microbial metabolic activity in T2DM pathophysiology and support the microbiota-modulating potential of sitagliptin.

At the functional level, several metabolic functional pathways, such as PRPP-PWY, PENTOSE-P-PWY, PANTO-PWY, PANTOSYN-PWY, SER-GLYSYN-PWY, PWY-7456, and GLYCOCAT-PWY, showed significant alterations between the ND and T2DM groups. Sitagliptin treatment effectively reversed these changes. Notably, PENTOSE-P-PWY and SER-GLYSYN-PWY have been previously linked to metabolic syndrome and T2DM (Li et al., 2023; Ge et al., 2020). GLYCOCAT-PWY is directly involved in glucose catabolism, highlighting a potential mechanism by which sitagliptin may exert metabolic benefits via the gut microbiota.

Moreover, analysis of gut microbial network parameters revealed significant alterations in the gut microbial community structure of T2DM rats, characterized by reduced connectivity and disrupted modular organization. These changes suggest a loss of microbial ecosystem stability commonly observed in metabolic disorders. Notably, sitagliptin treatment restored network complexity and increased interactions, which may contribute to improved gut barrier function and reduced systemic inflammation. These findings align with emerging evidence that microbial network integrity plays a critical role in host metabolic homeostasis (Huang et al., 2023). Future studies should focus on identifying key microbial “hub” species and their functional contributions, which could serve as novel therapeutic targets in T2DM management.

Interestingly, sitagliptin administration in ND rats (ND-Sit group) led to a significant reduction in the number of observed features and a downward trend in the Shannon index compared to the untreated ND group. One possible explanation is that DPP-4 inhibitors such as sitagliptin can influence host–microbiota interactions by modulating incretin levels, gut hormone secretion, and intestinal motility, which in turn could affect microbial diversity. Previous studies have shown that GLP-1, which is upregulated by DPP-4 inhibition, may impact gut barrier function and microbial ecology even in healthy subjects (Zhang et al., 2019; Zeng et al., 2024). The reduction in alpha diversity in the ND-Sit group might reflect a selective pressure favoring certain bacterial taxa, potentially resulting in a more specialized but less diverse microbial community. Although reduced alpha diversity is often considered detrimental. In this study, the change in diversity did not appear to cause adverse effects on host physiology, as no significant differences in weight, behavior, or gut histology were observed in ND-Sit rats. Nevertheless, these findings highlight the need for further investigation into the effects of DPP-4 inhibitors on the gut microbiome of healthy individuals and underscore the importance of careful interpretation of microbiota changes in pharmacological studies.

While our findings demonstrate that sitagliptin modulates gut microbial composition and improves intestinal barrier integrity, the underlying molecular or cellular mechanisms contributing to these extra-glycemic effects remain largely unclear. Sitagliptin is known to inhibit DPP-4, an enzyme expressed not only in plasma but also in intestinal epithelial and immune cells, suggesting potential local effects within the gut (Yan et al., 2016). Emerging evidence indicates that DPP-4 inhibitors may influence gut permeability, inflammation, and microbial interactions through GLP-1-mediated pathways, modulation of immune cell activity, or preservation of epithelial homeostasis independent of glycemic control (Zhang et al., 2019). In addition, enhanced levels of GLP-1 may exert direct effects on gut epithelial cells and mucosal immunity, contributing to improved barrier function (Zeng et al., 2024). Future studies are warranted to explore these mechanisms using targeted assays and pathway analyses to better delineate the extra-glycemic actions of sitagliptin.

Despite these promising findings, several limitations should be acknowledged. This study employed 16S rRNA gene sequencing with functional predictions based on PICRUSt2, which, while informative, require validation through direct metabolomic and metatranscriptomic analyses, and metagenomic sequencing for a deeper exploration of microbial functions and interactions at species or strain levels. The observed effects in the rodent model also warrant confirmation in human cohorts and mechanistic exploration using germ-free or transgenic animal models. To more comprehensively evaluate the protective effects of sitagliptin on gut epithelial integrity, future studies should incorporate direct permeability assays such as the FITC-dextran test. Moreover, the lack of measurements of key inflammatory markers (e.g., serum LPS) and circulating GLP-1 levels limits the interpretation of systemic inflammation and GLP-1–mediated mechanisms. The sitagliptin intervention period was limited to 12 weeks, and it remains unclear whether the observed benefits on gut microbiota and barrier function are sustained long-term. Future studies with prolonged treatment durations and comparative interventions, such as metformin, will be necessary to clarify the durability and specificity of these effects.

In summary, this study provides novel evidence that sitagliptin not only improves glycemic control but also exerts restorative effects on gut microbiota composition, diversity, and function, as well as intestinal barrier integrity in a T2DM rat model. These findings highlight the potential of sitagliptin as a therapeutic agent with multifaceted benefits, underscoring the critical role of the gut microbiome in the pathogenesis and treatment of type 2 diabetes mellitus.

## Data Availability

The datasets presented in this study is publicly available. This data can be found here: https://www.ncbi.nlm.nih.gov/, accession PRJNA601240.
